# Nutritional Profiling and Labeling Practices of Plant-Based, Hybrid, and Animal-Based Dog Foods: A Study of European Pack Labels (2020–2024)

**DOI:** 10.3390/ani15131883

**Published:** 2025-06-26

**Authors:** Fatma Boukid, Kurt A. Rosentrater

**Affiliations:** 1 ClonBio Group Ltd., 6 Fitzwilliam Pl, D02 XE61 Dublin, Ireland; 2 Agricultural and Biosystems Engineering, Iowa State University, Ames, IA 50011, USA; karosent@iastate.edu

**Keywords:** pet food, nutritional adequacy, labeling, plant-based, sustainability, pricing trends

## Abstract

As pet owners become increasingly mindful of pet health and sustainability, labeling plays a crucial role in shaping informed purchasing decisions for pet food. This study evaluated the nutritional adequacy and pricing of plant-based, hybrid, and animal-based dog foods. Using the Mintel database, we analyzed product labels, ingredient compositions, and marketing claims for various dog food categories. The findings revealed notable differences in key nutrients, such as protein, fiber, fat, ash, and moisture content. Plant-based dog foods generally offer higher fiber and ash content but often fall short in protein and fat levels, particularly in snacks and treats, which may impact their suitability for meeting the dietary needs of canines. Hybrid dog foods, which blend plant and animal ingredients, show greater variability, with some achieving balanced protein and fat content, while fiber levels depend on the plant-based proportion. Animal-based foods tend to excel in protein and fat content, particularly in wet and dry formats, while being lower in fiber and ash content. A key concern is the reliance on additives, particularly in plant-based and hybrid options, which raises questions about the long-term health impacts on pets. Pricing trends indicate that plant-based dog foods are generally more expensive than hybrid and animal-based options, although the cost varies widely across all categories.

## Simple Summary

With a growing interest in pet health and environmentally friendly choices, many dog owners are exploring alternatives to traditional meat-based dog food. This study examined the labeling of plant-based, hybrid (plant and meat combined), and animal-based dog foods and compared their nutritional content and prices. We reviewed the labels of several dog food products sold in Europe between 2020 and 2024. We found that plant-based dog foods often have more fiber and minerals but usually contain less protein and fat, which are important for dogs’ health. Hybrid foods vary significantly depending on the amount of meat they contain, while traditional animal-based foods usually provide more protein and fat, especially in wet and dry types. We also noticed that plant-based and hybrid foods often include many additives, which might raise concerns regarding long-term dog health. In terms of cost, plant-based dog foods tend to be more expensive than other dog foods. These findings can help dog owners make better decisions and encourage pet food companies to improve their labeling and nutrition.

## 1. Introduction

The global pet food industry has undergone a significant transformation in recent years, driven by shifting consumer preferences, increasing environmental concerns, and advancements in pet nutrition science [1,2]. As pets are increasingly regarded as integral family members, pet owners are paying closer attention to the origin, composition, and sustainability of the food they provide [3,4]. This heightened awareness reflects broader global trends toward sustainable and ethical consumption, influencing the pet food market to evolve beyond traditional animal-based diets [5,6].

Although grain byproducts (like corn or wheat middlings) have long been used in dry pet food (kibble) formulations to provide carbohydrates, fiber, and sometimes cost-effective protein sources, commercial pet food, particularly dry kibble, has historically been a blend of animal- and plant-based ingredients since its inception over a century ago (e.g., James Spratt’s dog biscuits in the 1860s often included meat, wheat, and vegetables) [7,8]. While marketing emphasis may have traditionally centered on animal-based protein sources (such as meat, fish, or poultry), recent diversification represents a more overt acknowledgment and strategic development of hybrid (which combines animal-derived ingredients with plant-based components) and plant-based options. This shift reflects broader trends toward sustainability and ethical sourcing, as well as a more explicit alignment of pet diets with the values of their owners [9]. These developments address the growing awareness of the environmental impact of food production and the changing expectations of pet owners, who increasingly view their pets as family members and seek diets that align with their values [10,11]. Animal-based pet food, which relies entirely on ingredients derived from livestock, remains a cornerstone of the industry. It is valued for its high protein content and compatibility with the natural dietary preferences of pets [12]. However, concerns regarding the environmental footprint of animal agriculture have prompted scrutiny of this category [13]. On the other hand, hybrid pet food has long been a staple in the market [14,15]. While initially driven by cost efficiency, hybrid formulations also offer a more sustainable alternative by reducing the environmental impact associated with livestock production by lowering the overall demand for animal protein [9,16]. By strategically balancing animal- and plant-based ingredients, hybrid formulations can supply essential nutrients to pets while decreasing greenhouse gas emissions, reducing land and water usage, and lessening energy inputs compared to traditional meat-based diets [17].

Plant-based pet foods, including vegetarian and vegan options, have gained momentum as a niche but rapidly growing segment [18]. These diets appeal to environmentally conscious consumers and those driven by ethical considerations, such as the desire to reduce reliance on animal agriculture. However, pet owners face significant challenges when deciding whether to feed their pets a plant-based diet, especially when addressing the nutritional needs of dogs [19,20]. Ensuring the nutritional adequacy of these diets is critical, as several studies have indicated that commercial plant-based pet foods often fail to meet the needs of dogs, despite manufacturers’ claims [9,19,21]. Thus, many pet owners, particularly vegans and vegetarians, experience a conflict between their ethical beliefs and the perceived need to provide their pets with a diet based on animal products [3,22,23]. However, the growing availability and increasing willingness of pet owners to purchase plant-based options, combined with increasing research on their nutritional suitability, may help resolve this dilemma.

Pet owners often rely on the information provided on product labels to make informed decisions [24]. Product labeling is crucial as it communicates essential details about the nutritional adequacy, ingredient composition, and feeding guidelines of pet foods. In the European Union, pet food labeling is regulated by EU Regulation No. 767/2009 and its subsequent amendments, which ensure transparency and prevent misleading claims. In addition to regulatory requirements, the European Pet Food Industry Federation (FEDIAF) plays a key role in supporting the pet food industry [25]. The FEDIAF provides comprehensive guidelines for pet food labeling and formulation, ensuring compliance with EU standards while promoting best practices in the industry. Their guidelines also help address the nutritional adequacy of pet foods by recommending nutrient levels based on the life stages and physiological needs of pets.

Proper product labeling is fundamental across all pet food categories; however, it receives particular consumer focus in plant-based options, given that consumers are increasingly scrutinizing claims of nutritional parity with traditional animal-based options [26]. Although plant-based dog products cater to ethical and environmental considerations, their ability to provide a complete and balanced diet remains a critical concern. In this light, this study focuses on the evolving landscape of dog food in the European market, including snacks, treats, wet food, and dry food. This study explores the growth of hybrid, plant-based, and traditional animal-based products over the past five years, focusing on their ingredient composition, nutritional adequacy, marketing claims, and pricing. It specifically evaluates whether plant-based options can match their animal-based counterparts.

## 2. Materials and Methods

### 2.1. Data Collection and Extraction

The Mintel Global New Product Database (Mintel GNPD; Mintel Group Ltd., London, UK) was used to select the dog products. An initial overview of product launches dating back to 1996 was conducted to contextualize the market trends within the dog food industry. For detailed data extraction and analysis, this study focused on dog products sold in Europe over the last five complete years (1 January 2020–31 December 2024), specifically from the “dog snacks and treats”, “wet dog food”, and “dry dog food” categories. In this study, products labeled as nutritionally complete (i.e., formulated to meet a dog’s full daily nutritional requirements) were classified as dog food (dry or wet), while products intended for occasional use such as for training, rewarding, or specific functions, were categorized as complementary products under “snacks and treats”. The database search will be conducted on 20 January 2025. The specific search criteria are summarized in Table 1, and the retrieved data were exported to Microsoft Excel (Microsoft Office, Redmond, WA, USA) for systematic review and analysis.

The products were categorized based on claims such as vegan/no animal ingredients, vegetarian, and plant-based. Ingredients and nutritional information, including fiber, protein, ash, moisture, and fat, were extracted and verified from product labels or packaging available in the Mintel database. These data were systematically reviewed to classify each product into one of three distinct categories based on the ingredient composition and macronutrient contribution:

- Animal-based products: Formulations in which animal-derived ingredients constitute the predominant proportion and serve as the principal sources of protein and fat. Minor inclusions of plant-derived components (e.g., cereals, starches, vegetables, and fruits) may be present primarily for functional purposes, such as binding, texture enhancement, palatability, or as incidental sources of micronutrients, without substantial contribution to the product’s key macronutrients. This category also includes products composed entirely of animal-derived ingredients, where identified.
- Hybrid products: Formulations characterized by the deliberate and significant integration of both animal- and plant-derived ingredients. Plant-derived constituents (e.g., legume proteins, cereals, and plant oils) substantially contribute to the primary macronutrient profile (protein, fat, or carbohydrate) alongside animal ingredients. These products represent a balanced blend in which neither animal nor plant sources solely dominate the functional nutrient composition.
- Plant-based products: Formulations composed exclusively of plant-derived ingredients (e.g., cereals, legumes, vegetables, fruits, and plant proteins) with no animal-derived components.

### 2.2. Ingredients Analysis

The ingredients of the selected products were carefully analyzed, focusing on the presence or absence of animal-derived components, plant-based ingredients, and any hybrid formulations. This involved extracting detailed ingredient lists from product labels and categorizing them accordingly.

### 2.3. Nutritional Composition

The nutritional composition (i.e., fiber, protein, ash, moisture, and fat) of each product was extracted from the product labels available in the Mintel GNPD. These components were selected for their fundamental role in assessing the overall nutritional profile of pet foods, as outlined in the FEDIAF guidelines. This study aimed to uncover variations and trends in the nutritional profiles of animal-based, hybrid, and plant-based products.

### 2.4. Claims

Claim information was extracted from product labels to identify how brands market their products in terms of dietary and ethical considerations. The claims were used to differentiate products into categories based on their positioning (sustainability, health, naturalness, plant-based, and demographics).

### 2.5. Price Sensitivity

Price data for the products (€/100 g of product) were extracted to understand price sensitivity with the product type, examining whether plant-based or hybrid products tend to be priced differently from animal-based products.

### 2.6. Statistical Analysis

Statistical analysis was conducted using the Statistical Package for Social Sciences software (IBM SPSS Statistics, Version 25.0, IBM Corp., Chicago, IL, USA). A univariate analysis of variance (ANOVA) was performed to assess the differences in the nutritional composition and price of dog food products. Post-hoc tests were applied to evaluate variations among animal-based, hybrid, and plant-based products, with statistical significance set at *p* ≤ 0.05. Boxplots were generated using SPSS. For a visual representation of the ingredients, WordClouds^®^ 2025 software (Rotterdam, The Netherlands) was utilized.

## 3. Results

### 3.1. Market Penetration and Product Launch Trend

**Figure 1:**
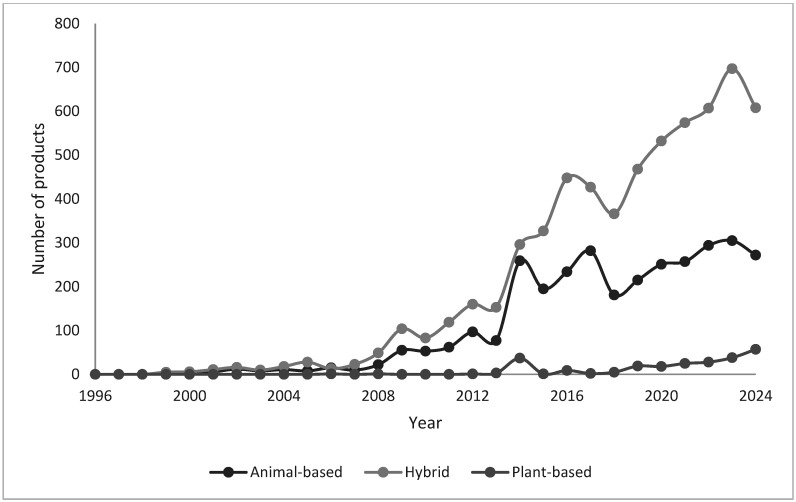
Evolution of the number of launches of animal-based, hybrid, and plant-based dog food products in the European market.

As shown in Figure 1, the dog pet food market from 1996 to 2005 was characterized by hybrid products with minimal animal- and plant-based options. During this period, the number of hybrid product launches was relatively low, ranging from 0 to 15 per year, with a peak of 15 in 2005. Animal-based products began to emerge in 2004 and 2005, with only one to three launches, while plant-based products were almost non-existent, with only one recorded in 2006.

The innovation and growth phases from 2006 to 2015 saw a significant shift. Hybrid products continued to grow consistently, with a sharp rise starting in 2010, peaking at 62 launches that year, and maintaining a strong presence throughout the phase. Animal-based research also increased steadily, especially from 2011 onward, growing from 49 launches in 2011 to 104 in 2014. Plant-based products remained rare, with only one or two launches per year, except in 2015, when five plant-based products were launched.

The expansion phase from 2016 to 2024 marks a period of considerable growth and diversification in the pet food market. Hybrid product launches have seen exponential growth, rising from 160 in 2016 to 608 in 2024, driven by increasing consumer demand for more balanced formulations combining animal- and plant-based ingredients. In 2024, 272 animal-based products were launched, a decrease from the peak of 282 in 2019. The plant-based product segment experienced the most significant growth, particularly post-2020, with launches surging from nine in 2020 to 57 in 2024. This trend reflects growing consumer interest in vegan, ethical, and environmentally sustainable pet food options.

By 2024, hybrid products still accounted for the largest share of launches (608), followed by animal-based (272) and plant-based (57) products, which showed the fastest growth.

The data presented in Table 2, which focuses on products launched between 2020 and 2024, illustrates some notable shifts in the types of products being introduced. Hybrid formulations have emerged as the dominant category in terms of new product launches, surpassing traditional animal- and plant-based formulations across all food types.

Hybrid products have seen the most significant increase, with 3018 products launched, accounting for 78.2% of all new products. This surge is especially prominent in the snacks and treats category, where hybrid products lead to 1855 launches. This trend highlights the growing consumer interest in products that offer a blend of animal and plant ingredients, catering to the desire for sustainable, ethical, and nutritionally balanced pet food options. The rise of hybrids reflects a broader shift in consumer preferences towards formulations that offer environmental and health benefits without fully abandoning the nutritional profile of traditional animal-based foods.

In comparison, animal-based products remain significant but represent a smaller share of recent launches, with 1379 products (35.4% of the total launches) showing slower growth than hybrids. Within the animal-based segment, most launches are found in snacks and treats (880 products), with fewer product introductions in wet food (479 products) and dry food (20 products). The slowdown in the growth of animal-based products may indicate a shift in consumer priorities, with factors such as sustainability and ethics increasingly influencing purchasing decisions.

Plant-based products, although the smallest category with 166 launches (3.6%), have also shown steady growth, especially in the snacks and treats (141 products) category. Their limited presence in wet (19) and dry food (6) formats may be attributed to technical and nutritional formulation challenges, as well as market hesitancy regarding the suitability of completely plant-based diets for dogs.

### 3.2. Ingredient Analysis

**Figure 2:**
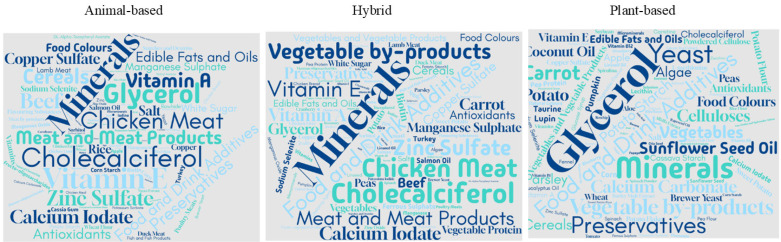
Word clouds representing the top 100 ingredients in dog food products. The ingredient size corresponds to the frequency.

Figure 2 illustrates the ingredients used in animal, hybrid, and plant-based dog food products. Tables S1–S3 provide detailed summaries of the most frequently included ingredients in each category, offering insights into their frequency and percentage presence across different product types.

For animal-based products (Table S1), food and drink additives dominated the ingredient list, with 761 occurrences, representing 55.2% of the total products examined. Among these additives, glycerol ranked second, with 440 mentions, comprising 31.9% of the products. Preservatives and antioxidants were also significant, appearing in 205 (14.9%) and 179 (13.0%) products. Other commonly found ingredients in this category include food colors, flavoring substances, and various preservatives, which appear less frequently but are still notable components in the formulation of animal-based products. Vitamins and minerals are equally prominent in the ingredient list. Cholecalciferol, a form of vitamin D, was found in 510 products, accounting for 37.0% of the total, followed by Vitamin E, which appeared in 407 products (29.5%). Other important minerals and vitamins included zinc sulfate (305 occurrences), calcium iodate (270 occurrences), and manganese sulfate (242 occurrences), with occurrences in 22.1%, 19.6%, and 17.6% of the products, respectively. Trace minerals, such as copper sulfate, iodine, and retinyl acetate, were also present but at lower frequencies, demonstrating the reliance on these micronutrients in animal-based food formulations. When considering meat and meat products, chicken meat was the most frequently used ingredient, appearing in 412 products (29.9%), followed by beef (270 occurrences, 19.6%) and lamb meat (93 occurrences, 6.7%). Other meats, including turkey, duck, and game meats, are less prevalent but still account for a portion of product formulations. Meat and meat products without any specification of origin were found in 558 products (40.5%), further indicating their importance in the formulation of animal-based products. Cereals and starches also contributed significantly to animal-based formulations, particularly rice, corn starch, and potato starch, which were included in 91 (6.6%), 92 (6.7%), and 83 (6.0%) products, respectively. Edible fats and oils were key components, with 168 occurrences (12.2%) across animal-based products. Salmon, linseed, and sunflower seed oils are some of the more commonly used fats, reflecting the incorporation of specific oils for nutritional and sensory attributes.

When examining hybrid products (Table S2), additives dominated this category, appearing in 2080 products (69.0%), showing a significant increase compared to animal-based formulations. Vitamins and minerals continue to play a crucial role, with cholecalciferol, vitamin E, and zinc sulfate being the most prevalent, appearing in 47.1%, 38.9%, and 27.6% of the products, respectively. Meat, particularly chicken meat, was also a central ingredient, featured in 982 (32.5%) hybrid products. Edible fats and oils, along with various preservatives, contribute to the formulation of these hybrid products. Vegetables were also more prevalent in the hybrid formulations (502 products, 16.6%). Plant proteins were reported at 16.7%, along with various legume ingredients (18.8% of total products).

Plant-based products (Table S3) have different compositions. Additives, including glycerol, antioxidants, and preservatives, are used in food and drink, appearing in 57.23% of the products. The use of vegetables such as carrots (42 products), potatoes (36 products), and sweet potatoes (36 products) is widespread, with fruits like apples, blueberries, and pomegranates also contributing to the formulations. Legumes and plant-based proteins, including peas (21 products), lupin (21 products), and pea protein (16 products), are essential components of many plant-based products, offering both nutritional value and texture. Fats and oils, such as coconut oil (33 products) and sunflower seed oil (26 products), also play a significant role in adding essential fatty acids and sensory appeal.

### 3.3. Nutritional Quality

#### 3.3.1. Snacks and Treats

**Figure 3:**
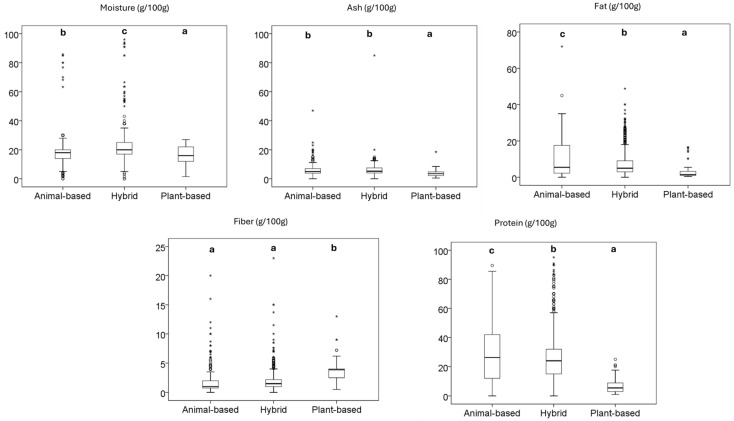
Nutritional facts of dog snacks and treats sold in the European market. Different letters indicate significant differences among them, as determined by the Tukey HSD test. The box represents the interquartile range (IQR), spanning from the first quartile (Q1) to the third quartile (Q3), with the median shown as a horizontal line. Whiskers extend to the minimum and maximum values within 1.5 times the IQR. Circles (○) denote mild outliers (1.5–3× IQR), and asterisks (*) indicate extreme outliers (>3× IQR).

The retrieved snacks and treats included various forms, such as sticks, bone-shaped biscuits, chews, protein snacks, and jerky, reflecting a diverse range of product types within this category. ANOVA revealed significant differences in the nutritional profiles of the three types of dog treats, focusing on moisture, ash, fat, fiber, and protein content (g/100 g) (Figure 3). Hybrid treats (21.59 g/100 g) showed higher moisture content than animal-based treats (18.75 g/100 g) and plant-based treats (16.45 g/100 g). Regarding ash content, both animal-based (5.77 g/100 g) and hybrid treats (5.81 g/100 g) had higher ash levels than plant-based treats (3.81 g/100 g). The fat content also differed significantly, with animal-based treats having the highest average fat content (9.71 g/100 g), followed by hybrid (7.66 g/100 g) and plant-based treats showing the least (2.96 g/100 g). In terms of fiber, plant-based treats had the highest content (3.74 g/100 g), while animal-based (1.75 g/100 g) and hybrid treats (1.90 g/100 g) contained much less. The protein content was highest in animal-based treats (30.50 g/100 g), followed by hybrid treats (26.28 g/100 g), and plant-based treats had the lowest protein content (6.93 g/100 g).

#### 3.3.2. Wet Food

**Figure 4:**
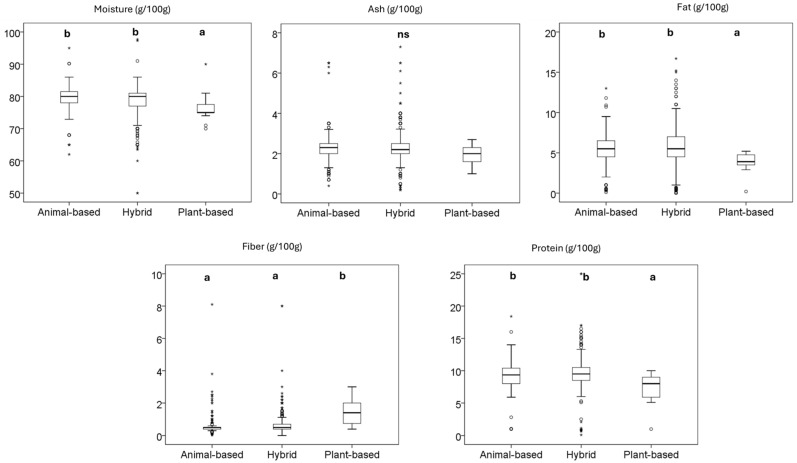
Nutritional facts of dog wet food products sold in the European market. Different letters indicate significant differences among them, as determined by the Tukey HSD test. The box represents the interquartile range (IQR), spanning from the first quartile (Q1) to the third quartile (Q3), with the median shown as a horizontal line. Whiskers extend to the minimum and maximum values within 1.5 times the IQR. Circles (○) denote mild outliers (1.5–3× IQR), and asterisks (*) indicate extreme outliers (>3× IQR). ns: not significant.

Based on Figure 4, wet dog foods showed significant differences in nutritional components (moisture, fat, fiber, and protein), but not in the ash content. Animal-based foods (79.56 g/100 g) had slightly higher moisture content than hybrid products (78.50 g/100 g), while plant-based products had the lowest moisture content (76.49 g/100 g). This aligns with the EDIAF recommendations for wet pet foods (60% and 84% moisture). Significant differences in fat content were observed, with animal-based and hybrid products having the highest fat content (5.55 g/100 g and 5.81 g/100 g, respectively). Plant-based products had significantly less fat (3.86 g/100 g), indicating a variation in fat content across product types. The fiber content was significantly higher in plant-based products (1.48 g/100 g) than in animal-based (0.55 g/100 g) and hybrid products (0.62 g/100 g). Protein levels were significantly higher in animal-based (9.17 g/100 g) and hybrid products (9.47 g/100 g), while plant-based products had the lowest protein content (7.31 g/100 g).

#### 3.3.3. Dry Food

**Figure 5:**
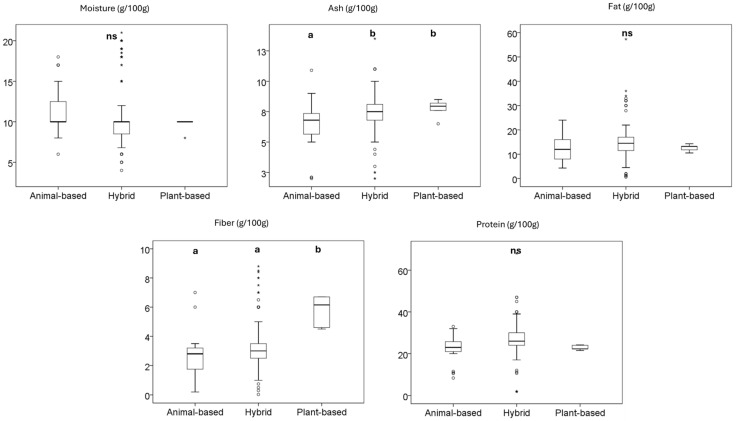
Nutritional facts of dog dry food products sold in the European market. Different letters indicate significant differences among them, as determined by the Tukey HSD test. The box represents the interquartile range (IQR), spanning from the first quartile (Q1) to the third quartile (Q3), with the median shown as a horizontal line. Whiskers extend to the minimum and maximum values within 1.5 times the IQR. Circles (○) denote mild outliers (1.5–3× IQR), and asterisks (*) indicate extreme outliers (>3× IQR). ns: not significant.

The nutritional profiles of the three types of dry dog food are presented in Figure 5. Significant differences were observed in moisture, ash, and fiber content, whereas moisture, fat, and protein content showed no notable variation. The moisture content of all categories ranged from 4 to 21 g/100 g, with an average value of 11.47 g/100 g for animal-based dry foods, followed by hybrid (10.86 g/100 g) and plant-based products (9.67 g/100 g). Regardless of the type, the moisture content was within the recommended range of FEDIAF for dry pet food (typically contains up to 14%). Plant-based and hybrid dry foods had the highest ash content, followed by animal-based dry foods. Plant-based products contained the highest fiber at 5.80 g/100 g, followed by hybrid products at 3.28 g/100 g, and animal-based products at 2.66 g/100 g. The average protein content values of plant-based (22.77 g/100 g), animal-based, and hybrid products (22.02 and 26.69 g/100 g) met the minimum recommended levels (18% protein for adults and 22% for puppies and lactating females) set by the FEDIAF.

### 3.4. Marketing Claims Analysis

The analysis of claims associated with dog food products in the European market reveals key trends that align with the evolving consumer preferences, market dynamics, and ethical considerations. These patterns are particularly evident in health, sustainability, and the distinct positioning of each product category, as shown in Tables S4–S6.

In the realm of snacks and treats (Table S4), health claims dominated the animal-based and hybrid product categories. Animal-based snacks frequently mention low/no/reduced fat (185 products) and grain-free options (174 products), catering to pet owners focused on weight management and food sensitivities. Hybrid snacks surpass these figures, with 455 products claiming low/no/reduced fat, 406 grain-free, and 530 with no added sugar, emphasizing their appeal to health-conscious consumers through the integration of animal- and plant-based ingredients. However, plant-based snacks showed a lower prevalence of health-related claims, with only 22 mentions for low/no/reduced fat and 53 for grain-free, suggesting a different marketing emphasis. Sustainability claims were most pronounced in hybrid snacks, with 349 products boasting environmentally friendly packaging and 305 emphasizing recycling. In contrast, animal-based snacks featured 125 claims for environmentally friendly packaging, while plant-based snacks contributed modestly with 50 and 27 claims for environmentally friendly and recyclable packaging, respectively. Plant-based snacks uniquely excel in plant-based ingredient claims, with 62 products labeled as vegetarian and 59 as vegan, reinforcing their distinct appeal to consumers seeking animal-free alternatives. Naturalness is another strong focus, where hybrid snacks lead with 843 products free from additives and 737 low in allergens. Animal-based snacks also highlight these attributes (384 and 336 products, respectively), while plant-based snacks emphasize clean labels, with 175 products free from artificial additives. Age-related targeting in snacks skews heavily toward adult pets across all categories, with hybrid products also addressing junior (100) and senior (15) stages, while plant-based options show minimal focus on life-stage differentiation.

In dry food (Table S5), hybrid products dominated health claims, with significant mentions of grain-free (80 products), vitamin/mineral fortification (137 products), and functional digestion support (115 products). In contrast, animal-based dry foodsexhibited limited health-related claims, such as one product labeled grain-free and seven products for digestion. Plant-based dry food, though niche, highlights health with four products claiming no added sugar and one for low/reduced sugar. Hybrid dry food also leads to functional health attributes like joint, bone, and muscle support (75 claims) and skin and coat benefits (100 claims). Sustainability claims were most prevalent in hybrid products, with 71 products offering environmentally friendly packaging and 62 promoting recycling. Animal-based dry food made modest contributions with seven claims each, while plant-based options consistently showed niche sustainability claims (five products for both categories). The distinct appeal of plant-based dry food is further emphasized through claims like vegetarian (5 products), vegan (1 product), and plant-based (4 products), which are absent in animal-based and hybrid products. Naturalness-related claims were dominated by hybrid products, with 145 products claiming no additives and/or preservatives. Animal-based dry food contributes fewer claims (10 and 14 products), while plant-based dry food emphasizes clean formulations with niche claims like free from artificial colorings (five products). Age-related targeting in dry food favors hybrid products, with 204 mentions for adult pets and broader coverage for junior (25 products) and senior (nine products) pets. Plant-based options remain niche, with only four adult claims and no presence in the junior or senior categories.

Hybrid wet foods (Table S6) also led to health-related claims, with 291 products promoting grain-free, 171 no added sugar, and 326 vitamin/mineral fortification. Animal-based wet foods followed with 104 grain-free claims and 132 vitamin/mineral fortified mentions. In contrast, plant-based wet food remains limited in health-related claims, such as seven products claimed to be fortified with vitamins and/or minerals, and two products for no added sugar claims. Functional health claims were similarly dominated by hybrid products, with 156 mentions for digestion support and 99 for joints, bones, and muscles. Plant-based wet foods offered minimal contributions, with only three products claimed to aid digestion and one claimed to provide immune system benefits. Sustainability claims align with the overall trend, with hybrid wet foods leading to environmentally friendly packaging (329 products) and recycling (278 products), while animal-based wet foods contribute 0 and 61 products, respectively, and plant-based wet foods maintain modest but consistent mentions (8 and 8). Plant-based wet foods stand out with unique claims like vegan/no animal ingredients (13), plant-based (6), and vegetarian (3). In terms of naturalness, hybrid wet foods led with 441 products claiming the absence of additives/preservatives and 500 products claiming to be low in allergen mentions. Animal-based wet foods followed with lower but substantial claims, while plant-based wet foods contributed minimally, with seven products having no additives/preservatives and 14 products having low allergens. Age-related claims in wet food highlighted hybrid dominance, with 678 products for adult pets, 59 for juniors, and 26 for seniors. Plant-based wet food remains a niche, with only 12 products with adult claims and no presence in junior or senior categories.

### 3.5. Pricing

The mean and standard deviation of the prices of dog food products across the three categories (animal-based, hybrid, and plant-based) were compared within each subcategory (Table 3). Plant-based snacks and treats were the most expensive (€3.17 ± 2.46/100 g), followed closely by animal-based products (€3.11 ± 3.09/100 g). Hybrid products were significantly less expensive (€2.59 ± 2.08/100 g) than other products. Similarly, significant price differences were also found in the wet food subcategory (*p* ≤ 0.001). Plant-based wet food had the highest mean price (€1.04 ± 0.49/100 g), significantly exceeding both hybrid (€0.64 ± 0.66/100 g) and animal-based product prices (€0.49 ± 0.39/100 g). No significant differences were detected in the dry-food category (*p* > 0.05). The mean prices were €0.95 ± 0.95/100 g for animal-based products, €1.00 ± 1.26/100 g for hybrid products, and €0.79 ± 0.17/100 g for plant-based products.

## 4. Discussion

The dog food market has significantly evolved from 1996 to 2024, marked by the emergence of plant-based and hybrid products alongside the traditional dominance of animal-based formulations. This shift reflects changing consumer preferences driven by growing awareness of sustainability, health, and ethics in pet food choices. Initially, animal-based products were the dominant segment, providing dogs with a familiar and biologically appropriate diet. However, as consumer demand for more sustainable and nutritionally diverse options has increased, hybrid and plant-based formulations have gained considerable traction. These trends align with broader societal movements towards ethical consumption and environmental sustainability [27].

The formulation of dog food plays a crucial role in ensuring that it meets the nutritional recommendations of the FEDIAF, which ensures that pet foods meet basic nutritional standards. An in-depth examination of animal-based, hybrid, and plant-based dog foods reveals distinct nutritional profiles, each with its own strengths and limitations. High variability was observed in all products, regardless of category, showing the versatility of the offering. Animal-based dog foods are known for their high-quality protein content derived from meat and meat byproducts such as chicken, beef, and lamb [28,29]. These protein sources are complete, providing all the essential amino acids in bioavailable forms, which are crucial for muscle growth, tissue repair, and overall health [30,31]. However, the environmental impact of livestock farming, particularly in terms of greenhouse gas emissions and land use, raises concerns regarding the sustainability of animal-based products [32]. Hybrid formulations, which blend animal and plant ingredients, typically offer comparable protein content and amino acid profiles to animal-based products [33,34]. While these hybrid foods comply with regulatory requirements, careful formulation is necessary to ensure that the amino acid profile is complete and bioavailable, especially when plant proteins comprise a significant portion of the formula [23]. Plant-based dog foods present a greater challenge in meeting dogs’ protein needs, a pattern particularly evident in snacks and treats, as well as wet foods, which exhibit significantly lower protein content than animal-based and hybrid options. For dry foods, while the protein content was numerically lower in plant-based products, all types met the minimum recommended levels. Although legumes, peas, and other sources (algae and yeast) can contribute to protein, these sources are often incomplete and have lower bioavailability than animal proteins [23,35]. Thus, formulating plant-based products with blends of proteins from different sources with complementary amino acid profiles might improve the quality of the protein to meet the needs of dogs [19,20,36]. Careful attention is needed to ensure that the protein content is adequate and well-balanced, as outlined by the FEDIAF recommendations (minimum of 12.5 g/MJ ME corresponding to around 18 g/100 g).

Fat content is another essential consideration in dog food formulation. Animal-based dog foods tend to be higher in fat, especially due to the inclusion of animal fats. Our analysis confirmed this trend for snacks and treats, where animal-based options exhibited the highest fat content and plant-based treats contained the least. Similarly, in wet foods, animal-based and hybrid products showed significantly higher fat contents than their plant-based counterparts. These fats are rich in omega-3 and omega-6 fatty acids, which are important for cognitive function, immune response, and skin health [37,38]. Hybrid formulations generally offer a more balanced fat content by incorporating animal and plant oils. Plant-based oils, such as flaxseed and canola oils, provide essential fatty acids, although they may not supply the same level of omega-3s as animal sources [39]. Hybrid products can offer a balanced fat composition, as they contain both animal- and plant-derived fats. Plant-based dog foods are typically lower in fat than animal-based dog foods, as plant ingredients provide less fat. Although vegetable oils contribute essential fatty acids, they may not provide the same concentration of omega-3 fatty acids [40]. Therefore, plant-based diets may need to be fortified with specific fatty acids, such as eicosapentaenoic acid (EPA) and docosahexaenoic acid (DHA), to meet dogs’ nutritional needs [41].

Fiber is an important nutrient for digestive health, helping to regulate bowel movements and support the gut microbiome [42]. Animal-based dog foods generally contain little or no fiber [43]. While this may not pose a problem for dogs with normal digestion, it can lead to digestive issues for some dogs, especially if the diet is not supplemented with fiber-rich ingredients [44]. Hybrid formulations benefit from the inclusion of plant-based ingredients, such as vegetables, legumes, and grains, which help increase fiber content. Our analysis aligns with these observations: plant-based treats exhibited the highest fiber content, while animal-based and hybrid treats contained considerably less. Similarly, plant-based wet and dry foods also showed significantly higher fiber levels than their animal-based and hybrid counterparts. Plant-based dog foods naturally contain higher fiber levels due to the reliance on plant ingredients like legumes, grains, and vegetables [45]. Studies have shown that fiber intake is beneficial for digestion and overall gut health, with fiber-rich diets improving gastrointestinal function and supporting a healthy gut microbiota [42,46]. Plant-based diets require careful balancing to ensure that fiber levels are appropriate and do not interfere with the absorption of other essential nutrients [47,48].

The ash content in dog food serves as a key indicator of the mineral composition of the product, representing inorganic components such as calcium, phosphorus, magnesium, and trace elements that are essential for a dog’s health [49,50]. Our study found that both animal-based and hybrid treats had higher ash levels than plant-based ones. For dry foods, plant-based and hybrid options also had the highest ash content compared to animal-based varieties. The higher ash content observed in animal-based and hybrid dog foods suggests a greater mineral load, which may be linked to the inclusion of animal-derived ingredients like bones, organ meats, and byproducts [51]. However, while these ingredients provide valuable nutrients, excess mineral levels can indicate poor-quality sources or excessive supplementation, raising concerns about the bioavailability and balance of minerals [52].

Claims play a significant role in consumer decision-making [27]. Plant-based dog products generally have a lower environmental footprint than meat-based formulations, aligning with consumer preferences for products that contribute to sustainability goals [53,54]. For plant-based products to succeed in the market, sustainability must be balanced with nutritional adequacy. Hybrid products present a more balanced approach by combining high-quality animal proteins with the sustainability benefits of plant ingredients [55]. This combination allows manufacturers to cater to both ethically and nutritionally focused consumers. However, the continued dominance of additives across all categories further underscores the complexity of producing pet foods that meet both naturalness-related expectations [29,56]. As consumer awareness of ingredient quality increases, plant-based products must evolve, reducing reliance on processed components while maintaining their ethical and environmental advantages [21,57].

Price is another important factor in consumer purchasing decisions [24,58]. Plant-based dog products often command higher prices due to the cost of sourcing specialty ingredients and the more complex formulation process required to balance plant-based proteins with essential nutrients [23]. Despite this, the relatively small price differences between plant-based and animal-based snacks and treats suggest that plant-based products are becoming more competitive and accessible as consumer demand increases. Hybrid products tend to fall between the prices of animal-based and plant-based foods, reflecting the balance between animal and plant ingredients in them. The greater variability in the pricing of snacks and treats reflects the diverse range of options available, from budget-friendly to premium offerings [59]. Although hybrid and plant-based products may initially seem more expensive, increasing competition in the market is likely to lead to more competitive prices as production processes are scaled up.

This study revealed several key facets. Examining the nutritional profiles of animal-based, hybrid, and plant-based dog foods from 2020 to 2024 provides valuable insights into current market trends, including the growing demand for more sustainable and nutritionally diverse pet food options. This study focused on key information on product labels, including nutritional information, claims, and ingredients. However, there are some limitations to this study. This study is constrained to protein, fiber, ash, and moisture contents, which means that details on vitamins, minerals, and amino acids, while essential to a complete nutritional profile, were not included in the analysis. Additionally, these data are based on product labels, which may not always fully represent the actual nutrient content due to variations in formulation and production processes. While the large sample size strengthens the study’s conclusions, the findings are specific to the products available during the 2020–2024 period. Therefore, the reliability of this study depends on the accuracy and completeness of the product labels, which could influence the overall validity of the conclusions.

## 5. Conclusions

In conclusion, the choice between animal-based, hybrid, and plant-based dog foods is driven by nutritional quality and marketing claims. This analysis highlights the key differences in nutrient composition, pricing, and positioning across the three categories. Plant-based dog foods are characterized by their higher fiber and ash contents but often fall short in protein and fat levels, particularly in snacks and treats. Hybrid dog foods exhibit varied nutrient profiles, with some achieving a more balanced protein-to-fat ratio. Animal-based products, particularly wet and dry foods, consistently provide higher protein and fat contents while having lower fiber and ash levels. Pricing trends reveal that plant-based dog foods are generally the most expensive, reflecting their reliance on specific supplemental ingredients and ethical considerations. Hybrid products occupy a mid-range price point. These findings emphasize the need for innovation in plant-based dog foods, particularly in enhancing protein and fat content while maintaining their fiber benefits and reducing costs.

Based on these findings, this study provides recommendations for both producers and consumers in the dog food sector.

For producers, further research and development are recommended to improve the nutritional profiles of plant-based dog foods, particularly by enhancing the protein and fat content and overall digestibility. This may include the use of novel plant protein sources and optimized processing techniques. Additionally, exploring cost-effective sourcing and manufacturing strategies may help address the relatively high prices of certain formulations. Improving transparency through accurate labeling and clear nutritional information can support informed decision-making and enhance consumer confidence. Product development should also consider the increasing demand for diets aligned with specific consumer values such as environmental sustainability, animal welfare, and dietary sensitivities.

For consumers, careful evaluation of product labels is essential to ensure nutritional adequacy, particularly in plant-based and hybrid products. Products should meet established nutritional standards (e.g., AAFCO or FEDIAF) and be appropriate for the dog’s life stage and health status. Consulting veterinarians or qualified pet nutritionists prior to major dietary changes is advisable, especially when introducing alternative protein sources. Consumers are also encouraged to consider the diversity within product categories and weigh factors such as ingredient composition, nutrient content, and price in relation to their dogs’ specific needs.

## Figures and Tables

**Table 1:** Categorization criteria for dog food products extracted from the Mintel database.

Categorization Criteria	Animal-Based Products	Hybrid Products	Plant-Based Products
Category	Dog food		
Sub-Categories (Format/Purpose)	Dog Snacks & Treats (Complementary) Wet Dog Food (Complete) Dry Dog Food (Complete)		
Region	Europe		
Date	1 January 2020, to 31 December 2024		
Claims	Not Vegan/No Animal Ingredients, Not Vegetarian, Not Plant-Based		Vegan/No Animal Ingredients, Vegetarian, Plant-Based
Ingredients	Not Vegetables and Vegetable Products (and all children)	Vegetables and Vegetable Products (and all children)	No animal ingredients
Nutrition	Fiber Protein Ash Moisture Fat		

**Table 2:** Launches of dog food products (expressed as number and percentage of the total product category) in the European market (2020–2024).

Category	Animal-Based	Hybrid	Plant-Based	Total Products
Snacks and treats	880 (30.6%)	1855 (64.5%)	141 (4.9%)	2876 (100%)
Wet food	479 (34.3%)	898 (64.3%)	19 (1.4%)	1396 (100%)
Dry food	20 (6.9%)	265 (91.1%)	6 (2.1%)	291 (100%)
Total	1379 (30.3%)	3018 (66.2%)	166 (3.6%)	4563 (100%)

**Table 3:** Mean and standard deviation of the prices of dog food products.

Category	Type	*p*-Value	Price (€/100 g)
Snacks and treats	Animal-based	***	3.11 ± 3.09 ^b^
	Hybrid		2.59 ± 2.08 ^a^
	Plant-based		3.17 ± 2.46 ^b^
Wet food	Animal-based	***	0.49 ± 0.39 ^a^
	Hybrid		0.64 ± 0.66 ^a^
	Plant-based		1.04 ± 0.49 ^b^
Dry food	Animal-based	ns	0.95 ± 0.95 ^a^
	Hybrid		1.00 ± 1.26 ^a^
	Plant-based		0.79 ± 0.17 ^a^

Different letters indicate significant differences among them, as determined by the Tukey HSD test. ns: not significant; ***: *p* ≤ 0.001.

## Data Availability

Data are available in the Supplementary Material.

## Supplementary Materials

The following supporting information can be downloaded at: https://www.mdpi.com/article/10.3390/ani15131883/s1, Table S1: Top ingredients in animal-based dog food products; Table S2: Top ingredients in hybrid dog food products; Table S3: Top ingredients in plant-based dog food products: frequency and percentage of inclusion; Table S4: Product claims in dog snacks and treats by type (animal-based, hybrid, and plant-based). Table S5: Claims in dog dry food products by type (animal-based, hybrid, and plant-based); Table S6: Claims in dog wet food products by type (animal-based, hybrid, and plant-based).
