# Supplementary material for: Nutritional Profiling and Labeling Practices of Plant-Based, Hybrid, and Animal-Based Dog Foods: A Study of European Pack Labels (2020–2024)
Source: Animals (Basel). 2025 Jun 26;15(13):1883. doi: 10.3390/ani15131883 (PMC12249005; doi:10.3390/ani15131883)
Supplement: Supplementary file 1 [file animals-15-01883-s001.zip › animals-3685147-supplementary.pdf]

Table S1: Top ingredients in animal-based dog food products.

Category	Ingredient	Number of products	% out of total products
**Additives**	Food and Drink Additives	761	55.2%
	Glycerol	440	31.9%
	Preservatives	205	14.9%
	Antioxidants	179	13.0%
	Food Colors	117	8.5%
	Flavoring Substances	78	5.7%
**Vitamins and Minerals**	Cholecalciferol	510	37.0%
	Vitamin E	407	29.5%
	Zinc Sulfate	305	22.1%
	Calcium Iodate	270	19.6%
	Manganese Sulphate	242	17.6%
	Vitamin A	230	16.7%
	Copper Sulfate	218	15.8%
	Manganese	87	6.3%
	Zinc	77	5.6%
	Retinyl Acetate	65	4.7%
	Iodine	64	4.6%
**Meat and Meat Products**	Meat and Meat Products	558	40.5%
	Chicken Meat	412	29.9%
	Beef	270	19.6%
	Lamb Meat	93	6.7%
	Duck Meat	83	6.0%
	Turkey	89	6.5%
	Poultry Meats	75	5.4%
	Deer Meat	51	3.7%
	Game Meat	35	2.5%
	Rabbit Meat	24	1.7%
**Cereals and Starches**	Cereals	226	16.4%
	Rice	83	6.0%
	Corn Starch	91	6.6%
	Potato Starch	92	6.7%
	Wheat	56	4.1%
	Cassava Starch	36	2.6%
	Rice Flour	35	2.5%
**Fats and Oils**	Edible Fats and Oils	168	12.2%
	Salmon Oil	56	4.1%
	Linseed Oil	47	3.4%
	Sunflower Seed Oil	34	2.5%
**Sugars and Sweeteners**	White Sugar	135	9.8%
	Sorbitol	88	6.4%
	Fructo-oligosaccharides	22	1.6%
**Other Ingredients**	Salt	198	14.4%
	Lignocellulose	66	4.8%
	Broth	29	2.1%
	Collagen	22	1.6%
	Biotin	102	7.4%
**Animal By-products**	Meat By-products	24	1.7%
	Beef Skin	59	4.3%

	Beef Heart	28	2.0%
	Beef Lung	23	1.7%
	Cattle Liver	35	2.5%
	Chicken Liver	42	3.0%
	Liver	38	2.8%
	Chicken Fat	26	1.9%
	Chicken Meal	102	7.4%
**Miscellaneous**	Natural Flavoring	89	6.4%
	Yucca Extract	98	7.1%
	Chelating Agent	29	2.1%
	E6 (Non-Food)	50	3.6%
	Tocopherol	30	2.2%
**Fish and Fish Products**	Fish and Fish Products	39	2.8%
	Cod	25	1.8%
**Eggs and Egg Products**	Eggs and Egg Products	26	1.9%

Table S2: Top ingredients in hybrid dog food products.

Category	Ingredient	Number of products	% out of total products
**Additives**	Food and Drink Additives	2080	69.0%
**Vitamins and Minerals**	Cholecalciferol	1422	47.1%
	Vitamin E	1172	38.9%
	Zinc Sulfate	834	27.6%
	Calcium Iodate	654	21.7%
	Copper Sulfate	707	23.4%
	Vitamin A	844	28.0%
	Manganese Sulphate	607	20.1%
	Biotin	381	12.6%
	Sodium Selenite	314	10.4%
	Vitamin B1	109	3.6%
	Vitamin B6	94	3.1%
	Vitamin C	107	3.5%
	Vitamin B12	87	2.9%
	Iodine	146	4.8%
**Meat and Meat Products**	Meat and Meat Products	1386	45.9%
	Chicken Meat	982	32.5%
	Beef	524	17.4%
	Lamb Meat	228	7.6%
	Duck Meat	199	6.6%
	Turkey	274	9.1%
	Poultry Meats	156	5.2%
	Chicken Liver	159	5.3%
	Chicken Breast	158	5.2%
**Cereals and Starches**	Cereals	635	21.0%
	Potato Starch	160	5.3%
	Corn Starch	113	3.7%
**Fats and Oils**	Edible Fats and Oils	654	21.7%
	Salmon Oil	276	9.1%
	Linseed Oil	145	4.8%
	Sunflower Seed Oil	144	4.8%
**Sugars and Sweeteners**	White Sugar	292	9.7%
	Sorbitol	171	5.7%
	Fructo-oligosaccharides	145	4.8%
**Vegetables and Vegetable Products**	Vegetables and Vegetable Products	502	16.6%
	Carrot	555	18.4%
	Vegetables	493	16.3%
	Sweet Potato	212	7.0%
	Potato	328	10.9%
	Spinach	127	4.2%
	Tomato	90	3.0%
**Plant-based proteins**	Vegetable Protein	504	16.7%
**Legumes**	Peas	398	13.2%
	Pea Protein	169	5.6%
**Other Ingredients**	Inulin	206	6.8%
	Salt	272	9.0%
	Glycerol	726	24.1%
	Preservatives	751	24.9%
	Antioxidants	630	20.9%

	Chelating Agent	168	5.6%
**Fruits**	Apple	201	6.7%
	Cranberry	155	5.1%
	Blueberry	112	3.7%
	Rosehip	89	2.9%
**Fish and Fish Products**	Fish and Fish Products	151	5.0%
	Salmon	161	5.3%
**Herbs and Spices**	Herbs	107	3.5%
	Rosemary	124	4.1%
	Chamomile	102	3.4%
	Nettle	132	4.4%
**Other**	Yucca	114	3.8%
	Glucosamine	121	4.0%
	Beetroot Pulp	119	3.9%
	Fish Fats	89	2.9%
	Algae	180	6.0%
**Miscellaneous**	Flavoring Substances	147	4.9%
	Yucca Extract	85	2.8%
	Copper Amino Acid Chelate	87	2.9%

Table S3: Top ingredients in plant-based dog food products: frequency and percentage of inclusion.

Category	Ingredient	Number of products	% out of total products
**Additives and Supplements**	Food and Drink Additives	95	57.23%
	Glycerol	67	40.36%
	Antioxidants	28	16.87%
	Preservatives	43	25.90%
	Celluloses	36	21.69%
	Vitamin E	21	12.65%
	Brewer Yeast	20	12.05%
	Cholecalciferol	20	12.05%
	Lecithin	13	7.83%
	Taurine	13	7.83%
	Sodium Selenite	12	7.23%
	Vitamins	7	4.22%
	Vitamin A	13	7.83%
	Vitamin B6	6	3.61%
	Vitamin B12	8	4.82%
**Vegetables and Vegetable Products**	Carrot	42	25.30%
	Vegetables	40	24.10%
	Parsley	35	21.10%
	Potato	36	21.69%
	Vegetable by-products	31	18.67%
	Vegetable Products	19	11.44%
	Pumpkin	24	14.46%
	Sweet Potato	17	10.24%
	Aloe	16	9.64%
	Fennel	14	8.43%
	Alfalfa Extract	10	6.02%
	Spinach	8	4.82%
	Broccoli	9	5.42%
	Zucchini	5	3.01%
	Tomato	7	4.22%
	Sweet Potato Flour	7	4.22%
	Lignocellulose	6	3.61%
**Fats and Oils**	Coconut Oil	33	19.88%
	Sunflower Seed Oil	26	15.66%
	Edible Fats and Oils	21	12.65%
	Linseed Oil	10	6.02%
	Rapeseed Oil	5	3.01%
**Legumes and Protein Sources**	Peas	21	12.65%
	Lupin	21	12.65%
	Pea Protein	16	9.64%
	Pea Flour	13	7.83%
	Soybean	8	4.82%
**Starches and Flours**	Potato Starch	37	22.29%
	Potato Flour	19	11.44%
	Cassava Starch	16	9.64%
	Wheat	11	6.63%
	Barley Malt Extract	11	6.63%
	Rice Flour	8	4.82%
	Corn	10	6.02%

	Corn Starch	6	3.61%
**Fruits**	Apple	22	13.25%
	Blueberry	10	6.02%
	Pomegranate	11	6.63%
	Strawberry	8	4.82%
	Rosehip	7	4.22%
**Herbs and Spices**	Eucalyptus Oil	15	9.04%
	Rosemary	7	4.22%
	Chamomile	7	4.22%
	Peppermint Oil	7	4.22%
	Turmeric	7	4.22%
	Basil	5	3.01%
	Thyme	5	3.01%
	Mint	5	3.01%
	Rosemary Extract	7	4.22%
**Seaweed and Algae**	Algae	21	12.65%
	Spirulina	5	3.01%
	Brown Algae	7	4.22%
**Fruit and Fruit Products**	Fruit and Fruit Products	6	3.61%
**Miscellaneous**	Natural Colors	9	5.42%
	Food Colors	7	4.22%
	Microminerals	8	4.82%
	Zinc Sulfate	9	5.42%
	Copper Sulfate	13	7.83%
	Zinc	6	3.61%
	Zinc Oxide	6	3.61%

Table S4: Product claims in dog snacks and treats by type (animal-based, hybrid, and plant-based).

Claim category	Claim	Animal-based	hybrid	Plant-based
Health	Low/no/reduced fat	185	455	22
	Grain-free	174	406	53
	No added sugar	171	530	40
	Vitamin/mineral fortified	116	420	14
	High/added protein	116	173	8
	Gluten-free	73	100	28
	Sugar-free	49	104	13
	Functional pet - joints, bones and muscles	37	95	7
	Added calcium	33	58	9
	Functional pet - skin and coat	29	123	6
	Functional pet - digestion	17	89	27
	Functional pet - immune system	15	84	8
	Dairy-free	14	64	10
	Low/no/reduced sodium	13	12	2
	Hypoallergenic	13	45	14
	Low/no/reduced lactose	12	7	1
	Low/no/reduced calorie	10	37	27
	Functional pet - brain and nervous system	10	9	2
	Functional pet - slimming	8	11	2
	Prebiotic	6	20	3
	Low/reduced sugar	4	1	0
	High/added fiber	3	4	7
	Functional pet - weight and muscle gain	3	8	0
Sustainability	Ethical - environmentally friendly package	125	349	50
	Ethical - recycling	116	305	27
	Ethical - environmentally friendly product	47	142	45
	Organic	23	12	10
	Carbon neutral	9	11	0
Plant-based	Vegetarian	0	0	62
	Vegan/No Animal Ingredients	0	0	59
	Plant-Based	0	0	42
	Ethical - Animal	18	30	14
Naturalness	No additives/preservatives	384	843	72
	Low/no/reduced allergen	336	737	102
	Free from added/artificial colorings	306	730	62
	Free from added/artificial flavorings	217	475	45
	Free from added/artificial preservatives	146	375	32
	All-natural product	117	123	17
	Free from added/artificial additives	59	89	175
	GMO-free	16	31	7
Age-related	Pet - adult	275	767	52
	Pet - junior	44	100	8
	Pet - senior	5	15	2

The numbers represent the count of products in each category that feature the specific claims between January 1st, 2020, and December 31st, 2024, as extracted from the Mintel database.

A single product may be counted multiple times if it features multiple claims.

Table S5: Claims in dog dry food products by type (animal-based, hybrid, and plant-based).

Claim category	Claim	Animal-based	hybrid	Plant-based
Health	Low/no/reduced fat	1	7	0
	Grain-free	1	80	1
	No added sugar	2	26	4
	Vitamin/mineral fortified	3	137	1
	High/added protein	2	42	0
	Gluten-free	3	37	0
	Sugar-free	2	13	0
	Functional pets - joints, bones and muscles	7	75	0
	Added calcium	0	114	0
	Functional pet - skin and coat	6	100	0
	Functional pet - digestion	7	115	0
	Functional pet - immune system	5	79	0
	Dairy-free	0	2	0
	Low/no/reduced sodium	0	2	0
	Hypoallergenic	3	24	0
	Low/no/reduced lactose	0	0	0
	Low/no/reduced calorie	1	2	0
	Functional pet - brain and nervous system	2	21	0
	Functional pet - slimming	2	11	0
	Prebiotic	0	57	0
	Low/reduced sugar	0	0	1
	High/added fiber	1	12	0
	Functional pet - weight and muscle gain	4	16	0
Sustainability	Ethical - environmentally friendly package	7	71	5
	Ethical - recycling	7	62	5
	Ethical - environmentally friendly product	5	49	5
	Organic	3	10	5
	Carbon neutral	3	9	0
Plant-based	Vegetarian	0	0	5
	Vegan/No Animal Ingredients	0	0	1
	Plant-Based	0	0	4
	Ethical - Animal	5	50	5
Naturalness	No additives/preservatives	10	145	4
	Low/no/reduced allergen	14	153	1
	Free from added/artificial colorings	9	122	5
	Free from added/artificial flavorings	7	97	5
	Free from added/artificial preservatives	5	97	0
	All-natural product	0	14	0
	Free from added/artificial additives	0	8	0
	GMO-free	5	51	0
Age-related	Pet - adult	15	204	4
	Pet - junior	1	25	0
	Pet - senior	1	9	0

The numbers represent the count of products in each category that feature the specific claims between January 1st, 2020, and December 31st, 2024, as extracted from the Mintel database.

A single product may be counted multiple times if it features multiple claims.

Table S6: Claims in dog wet food products by type (animal-based, hybrid, and plant-based).

Claim category	Claim	Animal-based	hybrid	Plant-based
Health	Low/no/reduced fat	5	23	2
	Grain-free	104	291	7
	No added sugar	70	171	2
	Vitamin/mineral fortified	132	326	7
	High/added protein	11	44	3
	Gluten-free	55	137	5
	Sugar-free	8	18	0
	Functional pet - joints, bones and muscles	47	99	2
	Added calcium	4	15	0
	Functional pet - skin and coat	58	166	2
	Functional pet - digestion	32	156	3
	Functional pet - immune system	27	112	1
	Dairy-free	0	23	0
	Low/no/reduced sodium	0	2	0
	Hypoallergenic	16	42	0
	Low/no/reduced lactose	1	12	0
	Low/no/reduced calorie	2	4	0
	Functional pet - brain and nervous system	5	2	0
	Functional pet - slimming	4	16	0
	Prebiotic	13	54	0
	Low/reduced sugar	0	0	0
	High/added fiber	2	13	1
	Functional pet - weight and muscle gain	2	15	0
Sustainability	Ethical - environmentally friendly package	0	329	8
	Ethical - recycling	61	278	8
	Ethical - environmentally friendly product	17	126	9
	Organic	23	63	8
	Carbon neutral	1	18	1
Plant-based	Vegetarian	0	0	3
	Vegan/No Animal Ingredients	0	0	13
	Plant-Based	0	0	6
	Ethical - Animal	58	99	6
Naturalness	No additives/preservatives	212	441	7
	Low/no/reduced allergen	210	500	14
	Free from added/artificial colorings	170	368	4
	Free from added/artificial flavorings	170	286	4
	Free from added/artificial preservatives	106	354	7
	All-natural product	19	100	0
	Free from added/artificial additives	13	27	2
	GMO-free	21	32	3
Age-related	Pet - adult	337	678	12
	Pet - junior	29	59	0
	Pet - senior	6	26	0

The numbers represent the count of products in each category that feature the specific claims between January 1st, 2020, and December 31st, 2024, as extracted from the Mintel database.

A single product may be counted multiple times if it features multiple claims.
